# White matter hyperintensity burden and infarct volume predict functional outcomes in anterior choroidal artery stroke: a multimodal MRI study

**DOI:** 10.3389/fnins.2025.1625882

**Published:** 2025-08-06

**Authors:** Weiwei Gao, Mingyang Wang, Jianzhong Lin, Junyi Huang, Lijuan Cai, Xingyu Chen, Renjing Zhu

**Affiliations:** ^1^Department of Neurology, Jimusaer County People's Hospital, Xinjiang, China; ^2^Department of Neurology, Zhongshan Hospital of Xiamen University, School of Medicine, Xiamen University, Xiamen, China; ^3^Xiamen Clinical Research Center for Cerebrovascular Diseases, Xiamen, China; ^4^Department of MRI, Zhongshan Hospital of Xiamen University, School of Medicine, Xiamen University, Xiamen, China; ^5^Department of Brain Science, Imperial College London, London, United Kingdom

**Keywords:** anterior choroidal artery, functional outcomes, infarction, infarct volume, white matter hyperintensity

## Abstract

**Objective:**

To investigate the relationship between white matter hyperintensity (WMH) burden and infarct volume with functional outcomes in patients with anterior choroidal artery (AChA) territory infarction.

**Methods:**

This retrospective cohort study included patients with AChA territory infarction admitted to two comprehensive stroke centers between September 2018 and September 2024. WMH burden was assessed using the Fazekas visual rating scale and an automated volumetric quantification method based on lesion prediction algorithms. Acute infarct volume was precisely measured using fully automated threshold segmentation. Poor functional outcome was defined as a modified Rankin Scale (mRS) score ≥3 at 90 days. Associations were evaluated using multivariable logistic regression models with stepwise adjustment for confounders, and predictive performance was assessed using receiver operating characteristic curve analysis. Restricted cubic spline (RCS) regression was employed to explore non-linear associations, followed by piecewise regression analysis based on threshold effects.

**Results:**

A total of 216 patients were included, of whom 73 (33.80%) had poor functional outcomes at 90 days. After adjusting for potential confounders, both WMH burden and infarct volume were independently associated with poor functional outcomes at 90 days, with infarct volume demonstrating superior predictive performance (AUC: 0.80 vs. 0.67). For each 1-mL increase in WMH volume, the risk of poor outcomes increased by 2% (adjusted OR = 1.02, 95% CI: 1.01–1.03, *p* = 0.032). RCS analysis revealed a non-linear association between infarct volume and poor outcomes, with a threshold of 2.7 mL. When infarct volume was below this threshold, each 1-mL increase in infarct volume was associated with a 5.31-fold increased risk of poor outcomes (adjusted OR = 5.31, 95% CI: 3.07–9.73; standardized OR = 3.03, 95% CI: 2.11–4.53).

**Conclusion:**

In patients with AChA territory infarction, both WMH burden and infarct volume can independently predict functional outcomes at 90 days. Infarct volume exhibits a non-linear association with outcomes, with a critical threshold of 2.7 mL identified.

## 1 Introduction

Stroke is the second leading cause of death and a major contributor to disability worldwide, posing a significant challenge to public health (Virani et al., 2020). According to a joint report by the World Stroke Organization and The Lancet Neurology, the global number of stroke-related deaths is projected to increase from 6.6 million to 9.7 million between 2020 and 2050, a staggering 50% increase. Moreover, the disability-adjusted life years attributable to stroke are expected to rise from 1.448 billion to 1.893 billion (Feigin et al., 2023). Among the various stroke subtypes, anterior choroidal artery (AChA) territory infarction accounts for 3.9–8.3% of ischemic strokes (Chausson et al., 2014; Cheng et al., 2020; Ois et al., 2009; Zhao et al., 2023). Despite its relatively low incidence, AChA infarction often leads to severe motor, sensory, and visual deficits due to the involvement of critical subcortical structures, such as the posterior limb of the internal capsule, cerebral peduncle, lateral thalamus, and lateral geniculate body, which serve as crucial pathways for motor and sensory conduction (Antoniello et al., 2023; Hupperts et al., 1994). These deficits significantly impact patients’ daily functioning and quality of life, placing a heavy burden on their families and society. Accurately assessing the prognostic risk of patients with AChA infarction and developing individualized management strategies accordingly are of great importance for improving patient outcomes and alleviating societal burden.

White matter hyperintensities (WMH) are a typical neuroimaging manifestation of cerebral small vessel disease in the elderly, with multiple pathophysiological processes implicated in their development, including chronic hypoperfusion, impaired cerebrovascular reactivity, blood–brain barrier disruption, oxidative stress, and inflammatory responses (Huang et al., 2024; Wu et al., 2021). Previous studies have established WMH burden as an independent predictor of 3-month functional outcomes, 1-year mortality, and long-term stroke recurrence in ischemic stroke (Bonkhoff et al., 2022; Derflinger et al., 2015; Dimaras et al., 2023; Gwak et al., 2024; Hong et al., 2021; Ryu et al., 2017; Ryu et al., 2019). However, the impact of WMH on prognosis may vary across stroke subtypes, and this association remains unclear, particularly in AChA territory infarction. Furthermore, existing studies have predominantly relied on visual rating scales for semi-quantitative assessment of WMH burden, which may not fully capture the spatial heterogeneity of WMH distribution due to their subjective nature, potentially underestimating the true association between WMH burden and outcomes. In addition to WMH, infarct volume is another crucial imaging predictor of prognosis. Studies have shown that AChA infarct volume positively correlates with the severity of neurological deficits and is closely associated with the risk of unfavorable outcomes (Chausson et al., 2014; Derflinger et al., 2015; Hupperts et al., 1994; Ois et al., 2009; Zhao et al., 2023). However, previous studies have largely relied on maximum lesion diameter measurements, which may not accurately reflect the spatial distribution and extent of tissue damage, limiting the accurate assessment of their predictive value.

To address these limitations, the present study employs a combination of quantitative volumetric measurements and visual rating scales to systematically evaluate the association of WMH burden and infarct volume with outcomes in AChA territory infarction. By enhancing the predictive accuracy of imaging markers, we aim to provide more reliable imaging-based evidence for early risk stratification and personalized treatment decision-making in this patient population.

## 2 Materials and methods

### 2.1 Study design and patient selection

This retrospective cohort study was conducted using electronic medical record databases from two comprehensive stroke centers in China. We consecutively enrolled patients admitted for acute AChA territory infarction between September 2018 and September 2023. The inclusion criteria were as follows: (1) age ≥18 years; (2) admission within 7 days of symptom onset; (3) acute ischemic lesion in the AChA territory confirmed by diffusion-weighted imaging (DWI); and (4) prestroke modified Rankin Scale (mRS) score ≤2. Patients were excluded if they met any of the following criteria: (1) concomitant acute infarcts in other arterial territories on DWI; (2) history of conditions that could affect the assessment of white matter signal, such as traumatic brain injury, brain tumors, infectious brain diseases, autoimmune brain diseases, toxic encephalopathy, or metabolic encephalopathy; (3) prior stroke with significant functional impairment or sequelae similar to the current clinical presentation, which could interfere with the evaluation of neurological function; and (4) incomplete follow-up data.

### 2.2 Data collection

We systematically collected the following baseline data: demographic information (age and sex), vascular risk factors [hypertension, diabetes mellitus, hyperlipidemia, coronary artery disease, atrial fibrillation, history of stroke/transient ischemic attack (TIA), smoking, and alcohol consumption], blood pressure at admission, and neurological function scores [National Institutes of Health Stroke Scale (NIHSS) and mRS scores at baseline and discharge]. Imaging characteristics, including lesion laterality, size, and involved anatomical regions, were recorded. Based on the maximum diameter on axial DWI, infarct lesions were classified as small (<20 mm) or large (≥20 mm). Additionally, data on intravenous thrombolysis and antiplatelet treatment strategies (no antiplatelet, single antiplatelet, or dual antiplatelet therapy) were collected.

All laboratory tests were performed on venous blood samples collected in the fasting state on the morning following admission, using standardized methods and calibrated equipment in the central laboratory of each hospital. The parameters assessed included complete blood count (white blood cells, neutrophils, lymphocytes, monocytes, red blood cells, hemoglobin, and platelets), biochemical tests (total protein, albumin, triglycerides, total cholesterol, low-density lipoprotein cholesterol, blood urea nitrogen, creatinine, and potassium), fasting blood glucose, and fibrinogen.

### 2.3 Imaging protocol

All patients underwent brain MRI within 24 h of admission using 1.5 T or 3.0 T magnetic resonance imaging systems (Siemens Healthcare, Erlangen, Germany). The scanning protocol included T1-weighted imaging (T1WI), T2-weighted imaging (T2WI), fluid-attenuated inversion recovery (FLAIR), and DWI. To ensure consistency of data between the two centers, uniform scanning parameters were used for FLAIR and DWI sequences. The parameters for FLAIR were as follows: repetition time (TR) 7,800 ms, echo time (TE) 89 ms, inversion time (TI) 2,500 ms, field of view (FOV) 256 mm × 256 mm, matrix 256 × 256, slice thickness 5.0 mm, interslice gap 1.5 mm, and 40 slices. The parameters for DWI were as follows: TR 3400 ms, TE 105 ms, FOV 160 mm × 160 mm, matrix 160 × 160, slice thickness 5.0 mm, interslice gap 1.5 mm, and 40 slices.

### 2.4 Imaging analysis

WMH burden and infarct volume measurements were performed collaboratively by two magnetic resonance imaging physicians (J. Lin and T. Kang) with more than 10 years of experience in stroke imaging diagnosis. All imaging analyses followed standardized protocols to ensure objectivity and reproducibility of measurements. All segmentation procedures relied solely on automated processing algorithms without manual correction or intervention to maintain objectivity and reproducibility.

**Figure 1:**
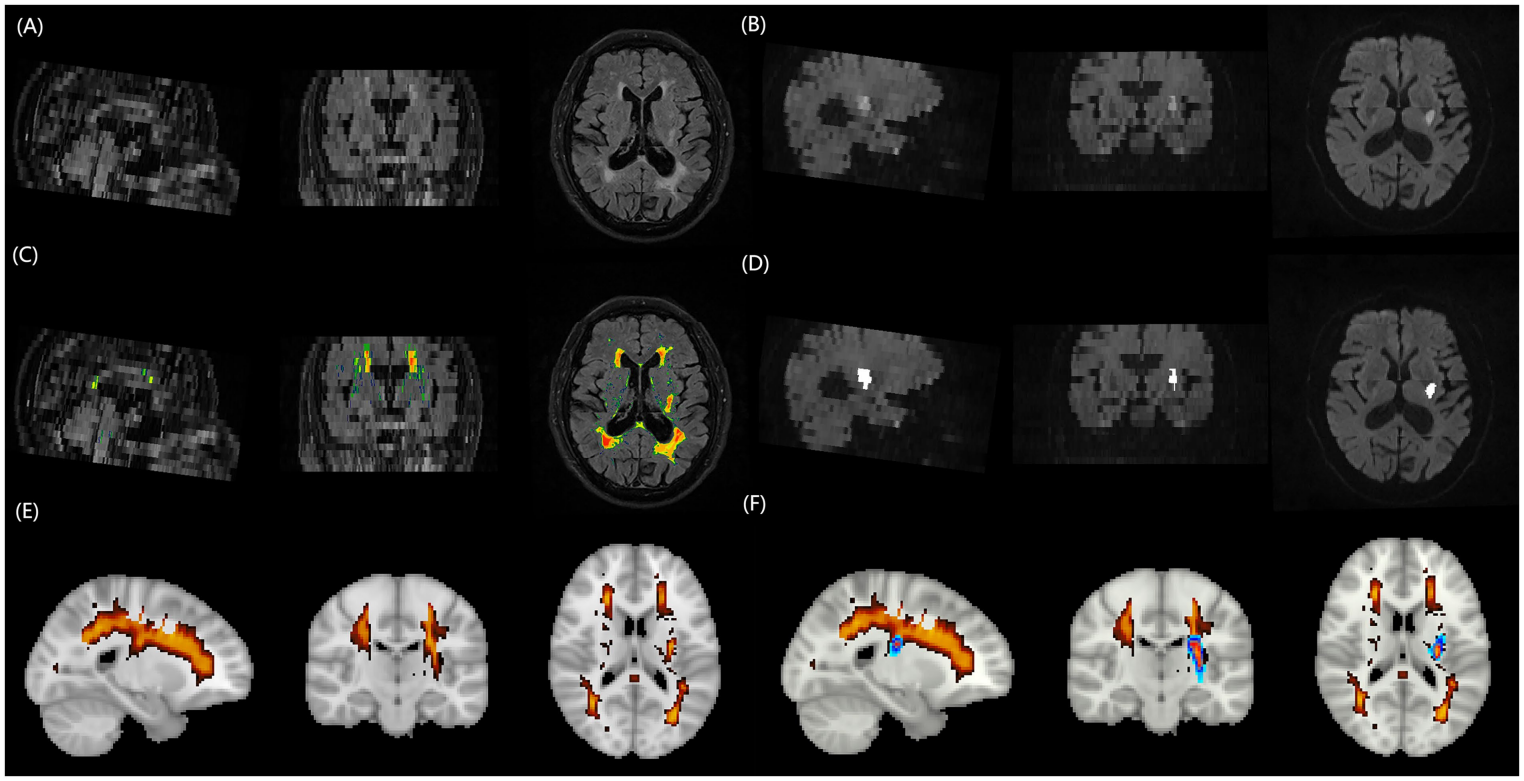
Schematic illustration of the multimodal MRI processing pipeline for quantifying WMH burden and acute infarct volume. **(A)** FLAIR image of a representative patient with AChA territory infarction, displaying extensive periventricular and deep WMH lesions. **(B)** DWI of the same patient, revealing an acute infarct in the right AChA territory. **(C)** Automated WMH segmentation result overlaid on the FLAIR image, generated using the lesion prediction algorithm from the Lesion Segmentation Toolbox in MATLAB. **(D)** Fully automated threshold segmentation result overlaid on the DWI image, obtained using a threshold-based approach. **(E)** Three-dimensional rendering of the segmented WMH lesions in the standard MNI152 space after spatial normalization, enabling the quantification of total WMH burden. **(F)** Three-dimensional rendering of the segmented infarct lesion in the standard MNI152 space, demonstrating the spatial relationship between the acute infarct (red) and WMH lesions (blue). MRI, magnetic resonance imaging; WMH, white matter hyperintensity; FLAIR, fluid-attenuated inversion recovery; DWI, diffusion-weighted imaging; AChA, anterior choroidal artery.

Quantitative analysis of WMH volume employed a fully automated segmentation method based on the lesion prediction algorithm (LPA). The analysis workflow comprised (Figure 1): (1) Image preprocessing: DICOM images were converted to NIfTI format using the dcm2niix tool; (2) Automated segmentation: Using the MATLAB platform, the LPA algorithm from the lesion segmentation toolbox (LST) was applied to automatically identify and segment WMH on FLAIR sequences^1^; (3) Quality control: Two experienced imaging physicians independently evaluated the automated segmentation results using a double-blind approach. The imaging datasets from both centers were randomized and anonymized prior to evaluation, with raters blinded to patient clinical information, outcomes, and hospital of origin. Each rater independently assessed the quality of automated segmentation results using a standardized 1–10 scoring system, where cases scoring <9 underwent re-segmentation after gamma transformation to optimize image contrast; (4) Spatial standardization: Rigid registration was performed using the FLIRT tool from the FSL software package, followed by non-linear registration algorithms to align individual images to MNI152 standard space, ultimately calculating standardized WMH volumes.

Acute infarct volume measurement employed fully automated threshold segmentation techniques to process DWI sequences. The processing steps included: (1) Skull stripping: FSL software was used to remove skull structures while preserving brain parenchyma; (2) Image standardization: Grayscale values were normalized to the 0–256 range, and adaptive histogram equalization techniques were applied to enhance contrast between lesions and normal brain tissue; (3) Threshold segmentation: Optimized threshold settings automatically identified diffusion-restricted regions to generate lesion masks; (4) Quality verification: Segmentation results were overlaid with original DWI images, with imaging physicians verifying segmentation accuracy and adjusting threshold parameters as necessary until satisfactory results were achieved.

Semi-quantitative assessment of WMH burden utilized the Fazekas visual rating scale. Based on anatomical location, WMH was classified into two subtypes: periventricular white matter hyperintensities (PVWMH) and deep white matter hyperintensities (DWMH). PVWMH scoring criteria were: 0 = absence of lesions, 1 = caps or pencil-thin lining, 2 = smooth halo, 3 = irregular periventricular hyperintensities extending into deep white matter. DWMH scoring criteria were: 0 = absence of lesions, 1 = punctate foci, 2 = beginning confluence of foci, 3 = large confluent areas. The total Fazekas score represented the sum of both subtype scores (0–6 points), based on which WMH severity was categorized as absent (0 points), mild (1–2 points), moderate (3–4 points), and severe (5–6 points). Fazekas scoring was performed independently by two experienced physicians (neurologist L. Cai and radiologist J. Lin), with raters blinded to patient clinical information. Scoring discrepancies were resolved through consensus meetings involving a third senior expert (T. Kang).

### 2.5 Imaging criteria for AChA territory infarction

The imaging diagnosis of AChA territory infarction was based on previously published criteria, which included the following (Antoniello et al., 2023; Hupperts et al., 1994): (1) the main body of the lesion (>2/3 to 1 of the volume) was located within the probabilistic distribution of the AChA territory^2^; and (2) the infarct area involved at least one of the following anatomical structures: the posterior 2/3 of the posterior limb of the internal capsule, the middle and posterior portions of the periventricular region (corona radiata), the lateral thalamus, the lateral geniculate body, the medial globus pallidus, the tail of the caudate nucleus, the medial temporal lobe, the hippocampus, and the basal 1/3 of the cerebral peduncle. Exclusion criteria included: (1) concomitant hemispheric infarction in the posterior limb of the internal capsule or periventricular region; (2) periventricular infarction extending beyond the posterior region (e.g., long-segment periventricular infarction or involvement of the subcortical region); (3) simultaneous infarction in the basal ganglia region and the posterior limb of the internal capsule; and (4) extensive infarction in the lentiform nucleus not limited to the posteromedial part.

### 2.6 Clinical outcomes

Functional outcomes were assessed using the mRS score. The primary outcome was functional status at 90 days after discharge, while the secondary outcome was neurological function at discharge. An mRS score of 0–2 was defined as a good outcome (functional independence), while a score of 3–6 was considered a poor outcome (functional dependence). All patients were followed up at 90 days after stroke onset either by telephone or in the outpatient clinic. The assessors underwent standardized training to ensure consistency and reliability of the evaluations.

### 2.7 Statistical analysis

Statistical analyses were performed using R software (Version 4.2.2, R Foundation for Statistical Computing, Vienna, Austria). The Shapiro–Wilk test was used to assess the normality of continuous variables. Normally distributed continuous variables were presented as mean ± standard deviation and compared between groups using the independent samples *t*-test. Non-normally distributed continuous variables were presented as median (interquartile range) and compared using the Mann–Whitney *U* test. Categorical variables were presented as frequencies (percentages) and compared using Pearson’s chi-square test or Fisher’s exact test.

**Figure 2:**
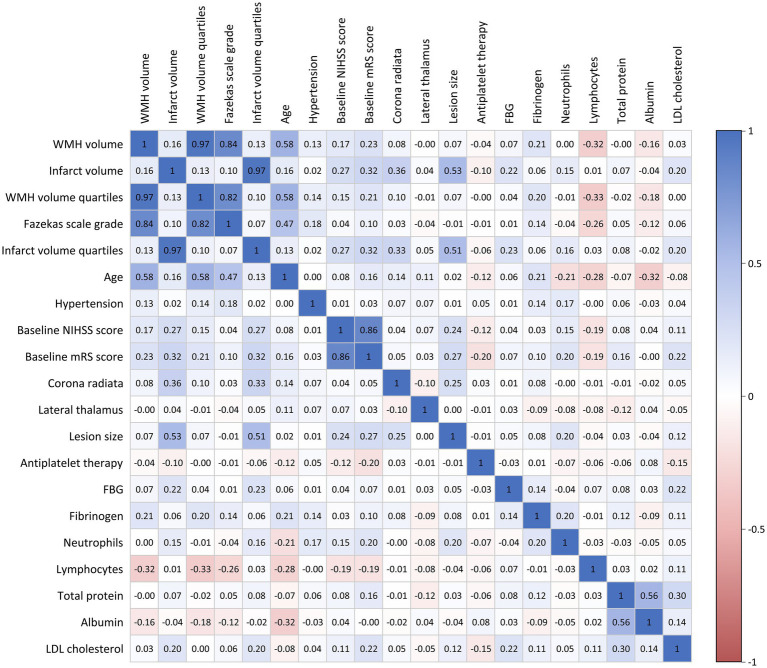
Correlation matrix of clinical variables and imaging parameters. Color intensity represents correlation strength: blue indicates positive correlations and red indicates negative correlations.

Prior to constructing multivariable logistic regression models, multicollinearity among candidate variables was assessed using Spearman correlation coefficient matrices, variance inflation factor (VIF), and tolerance values. Variables with correlation coefficients |*r*| > 0.7, VIF ≥ 10, or tolerance ≤ 0.1 were excluded from the analysis (Figure 2 and Supplementary Table S1). To investigate the associations of WMH burden and infarct volume with functional outcomes, we categorized WMH volume and infarct volume into quartiles and entered them into multivariable logistic regression models with stepwise adjustment for confounders. Model 1 was an unadjusted model; Model 2 was adjusted for age, hypertension, baseline NIHSS score, baseline mRS score, lesion size, involved anatomical regions (corona radiata and lateral thalamus), and antiplatelet treatment strategy; Model 3 further adjusted for laboratory parameters, including neutrophil count, lymphocyte count, total protein, albumin, low-density lipoprotein cholesterol, fasting blood glucose, and fibrinogen, in addition to the variables in Model 2. Furthermore, we constructed receiver operating characteristic (ROC) curves and calculated the area under the curve (AUC), sensitivity, and specificity. The DeLong test was used to compare the statistical differences in AUC values between different predictors.

Restricted cubic spline (RCS) regression models were used to assess potential non-linear associations between WMH burden, infarct volume, and functional outcomes at 90 days. We set four knots at the 5th, 35th, 65th, and 95th percentiles to construct the RCS functions. After determining the thresholds, continuous variables were standardized, and piecewise regression analysis was performed based on the thresholds. All variables with statistical significance (*p* < 0.05) in the univariate analysis were included as covariates in the multivariable analyses. All statistical tests were two-sided, and *p* < 0.05 was considered statistically significant.

## 3 Results

### 3.1 Patient characteristics

A total of 216 patients with AChA territory infarction were included in this study (Table 1). Among them, 155 (71.76%) were male, and the mean age was 63 years. Based on the 90-day mRS scores, patients were divided into two groups: good outcome (mRS 0–2, *n* = 143) and poor outcome (mRS 3–6, *n* = 73). Patients in the poor outcome group were significantly older than those in the good outcome group (*p* < 0.001) and had a higher prevalence of hypertension (*p* = 0.027).

**Table 1:** Baseline characteristics and clinical outcomes of patients with AChA territory infarction stratified by 90-day functional outcome.

Variables	Overall *N* = 216	Poor outcome *N* = 73	Good outcome *N* = 143	*p*-value
Age, years	63 ± 13	67 ± 14	61 ± 13	**<0.001**
Sex, male	155 (71.76)	47 (64.38)	108 (75.52)	0.085
Current smoker	77 (35.65)	24 (32.88)	53 (37.06)	0.543
Alcohol consumption	42 (19.44)	12 (16.44)	30 (20.98)	0.425
Medical history				
Hypertension	164 (75.93)	62 (84.93)	102 (71.33)	**0.027**
Diabetes mellitus	54 (25.00)	19 (26.03)	35 (24.48)	0.803
Hyperlipidemia	78 (36.11)	24 (32.88)	54 (37.76)	0.480
Atrial fibrillation	4 (1.85)	1 (1.37)	3 (2.10)	>0.999
Previous stroke/TIA	38 (17.59)	11 (15.07)	27 (18.88)	0.486
Coronary artery disease	19 (8.80)	4 (5.48)	15 (10.49)	0.219
Systolic blood pressure, mmHg	150 (138, 166)	147 (135, 169)	153 (141, 166)	0.675
Diastolic blood pressure, mmHg	90 (81, 99)	89 (81, 97)	91 (80, 100)	0.299
Baseline NIHSS score	3.0 (2.0, 6.0)	6.0 (3.0, 9.0)	2.0 (1.0, 4.0)	**<0.001**
Baseline mRS score	2.00 (1.00, 4.00)	4.00 (3.00, 5.00)	1.00 (1.00, 3.00)	**<0.001**
Lesion side				0.414
Left	107 (49.54)	39 (53.42)	68 (47.55)	
Right	109 (50.46)	34 (46.58)	75 (52.45)	
Lesion size				**<0.001**
Small infarcts (<20 mm)	173 (80.09)	45 (61.64)	128 (89.51)	
Large infarcts (≥20 mm)	43 (19.91)	28 (38.36)	15 (10.49)	
Anatomical involvement				
Posterior limb of internal capsule	170 (78.70)	62 (84.93)	108 (75.52)	0.110
Corona radiata	157 (72.69)	60 (82.19)	97 (67.83)	**0.025**
Medial globus pallidus	14 (6.48)	5 (6.85)	9 (6.29)	>0.999
Tail of caudate nucleus	23 (10.65)	10 (13.70)	13 (9.09)	0.299
Lateral thalamus	25 (11.57)	4 (5.48)	21 (14.69)	**0.045**
Lateral geniculate body	4 (1.85)	0 (0.00)	4 (2.80)	0.303
Medial temporal lobe/hippocampus	10 (4.63)	4 (5.48)	6 (4.20)	0.736
Cerebral peduncle	3 (1.39)	1 (1.37)	2 (1.40)	>0.999
Intravenous thrombolysis	39 (18.06)	16 (21.92)	23 (16.08)	0.292
Antiplatelet therapy				**0.007**
No antiplatelet	6 (2.78)	2 (2.74)	4 (2.80)	
Single antiplatelet	74 (34.26)	35 (47.95)	39 (27.27)	
Dual antiplatelet	136 (62.96)	36 (49.32)	100 (69.93)	
Discharge NIHSS	3.0 (1.0, 5.0)	6.0 (5.0, 9.0)	2.0 (1.0, 3.0)	**<0.001**
Discharge mRS	2.00 (1.00, 4.00)	4.00 (4.00, 5.00)	1.00 (1.00, 2.00)	**<0.001**
90-day mRS	2.00 (1.00, 3.00)	4.00 (3.00, 5.00)	1.00 (0.00, 1.50)	**<0.001**

Values are presented as n (%), mean ± SD, or median (IQR) as appropriate. NIHSS, National Institutes of Health Stroke Scale; mRS, modified Rankin Scale; TIA, transient ischemic attack; IQR, interquartile range; SD, standard deviation. Bold *p*-values indicate statistical significance (*p* < 0.05).

At admission, the poor outcome group had significantly higher NIHSS scores (*p* < 0.001) and mRS scores (*p* < 0.001) compared to the good outcome group. Analysis of imaging characteristics revealed that the proportion of large infarcts (≥20 mm) was significantly higher in the poor outcome group than in the good outcome group (*p* < 0.001). Regarding the anatomical distribution of lesions, the poor outcome group had a higher percentage of corona radiata involvement (*p* = 0.025), while the good outcome group had a higher percentage of lateral thalamus involvement (*p* = 0.045). Additionally, patients in the good outcome group more frequently received dual antiplatelet therapy compared to those in the poor outcome group (*p* = 0.007). There was no significant difference in intravenous thrombolysis between the two groups.

### 3.2 Laboratory findings

Laboratory test results (Table 2) showed that, compared to the good outcome group, the poor outcome group had significantly higher neutrophil counts (*p* = 0.005) and lower lymphocyte counts (*p* = 0.024). Regarding metabolic indicators, the poor outcome group had higher fasting blood glucose (*p* = 0.032) and low-density lipoprotein cholesterol levels (*p* = 0.011). Furthermore, the poor outcome group had elevated total protein (*p* = 0.015) and fibrinogen (*p* = 0.002) levels, while albumin levels were lower (*p* = 0.033).

**Table 2:** Laboratory parameters in patients with AChA territory infarction stratified by 90-day functional outcome.

Variables	Overall *N* = 216	Poor outcome *N* = 73	Good outcome *N* = 143	*P*-value
White blood cell, ×10^9^/L	4.72 (3.48, 6.11)	7.34 (6.31, 8.88)	6.92 (5.71, 8.69)	0.075
Neutrophils, ×10^9^/L	4.72 (3.48, 6.11)	5.01 (4.08, 6.45)	4.58 (3.31, 5.90)	**0.005**
Lymphocytes, ×10^9^/L	1.71 (1.33, 2.10)	1.56 (1.21, 2.02)	1.73 (1.43, 2.15)	**0.024**
Monocytes, ×10^9^/L	0.42 (0.34, 0.55)	0.44 (0.34, 0.56)	0.42 (0.35, 0.55)	0.632
Red blood cells, ×10^12^/L	4.63 (4.32, 4.94)	4.62 (4.32, 5.01)	4.63 (4.34, 4.92)	0.921
Hemoglobin, g/L	143 (131, 151)	139 (129, 149)	144 (134, 152)	0.178
Platelets, ×10^9^/L	231 (190, 268)	241 (198, 289)	224 (187, 259)	0.072
Total protein, g/L	70 (66, 74)	72 (67, 77)	69 (66, 73)	**0.015**
Albumin, g/L	41.1 ± 3.4	40.4 ± 3.5	41.4 ± 3.2	**0.033**
Triglycerides, mmol/L	1.35 (0.97, 2.04)	1.22 (0.96, 1.92)	1.37 (0.98, 2.32)	0.216
Total cholesterol, mmol/L	5.10 ± 1.21	5.23 ± 1.27	5.03 ± 1.17	0.264
LDL cholesterol, mmol/L	3.21 (2.70, 3.78)	3.46 (2.86, 3.97)	3.14 (2.65, 3.64)	**0.011**
BUN, mmol/L	5.19 (4.29, 6.38)	5.70 (4.40, 6.60)	5.07 (4.25, 5.90)	0.087
Creatinine, μmol/L	72 (63, 82)	72 (63, 85)	72 (62, 81)	0.622
Potassium, mmol/L	3.85 ± 0.36	3.81 ± 0.37	3.87 ± 0.35	0.305
FBG, mmol/L	5.76 (5.10, 7.01)	6.28 (5.21, 7.31)	5.53 (5.06, 6.90)	**0.032**
Fibrinogen, g/L	3.06 (2.58, 3.60)	3.37 (2.74, 3.87)	2.96 (2.52, 3.42)	**0.002**

Values are presented as median (IQR) or mean ± SD as appropriate. BUN, blood urea nitrogen; LDL, low-density lipoprotein; FBG, fasting blood glucose; IQR, interquartile range; SD, standard deviation. Bold *p*-values indicate statistical significance (*P* < 0.05).

### 3.3 WMH burden and infarct volume analysis

**Figure 3:**
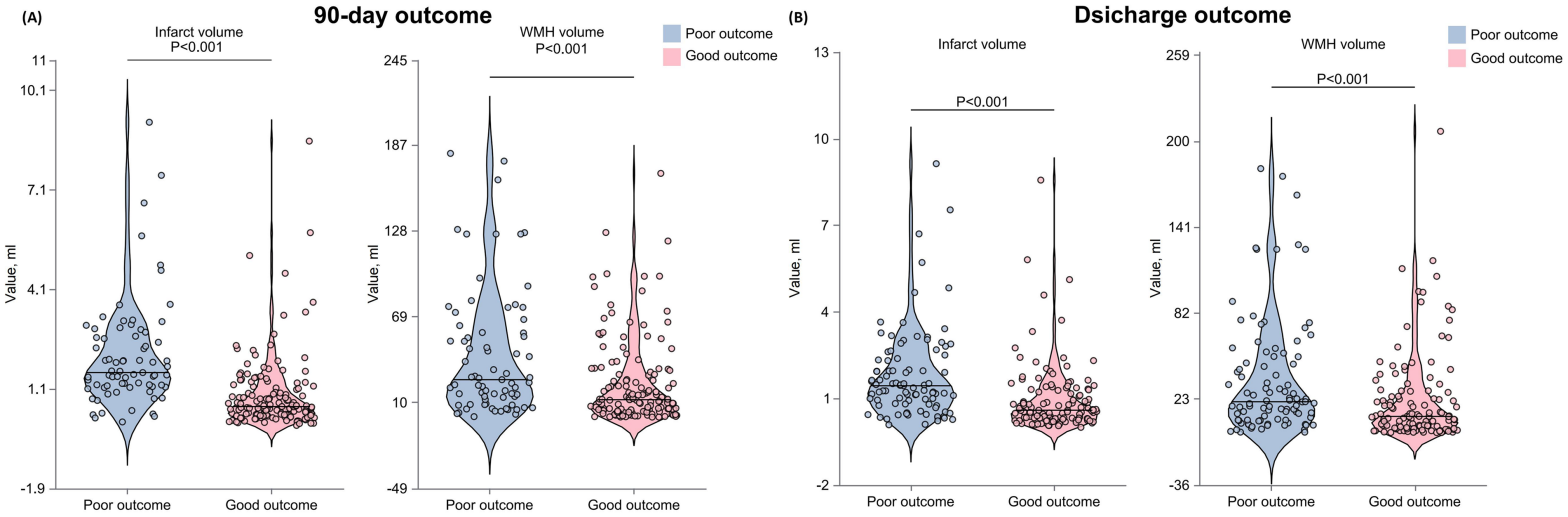
Distribution of WMH volume, infarct volume, and functional outcomes across quartiles at 90 days and discharge. The figure presents stacked bar charts displaying: the proportion of patients in each WMH volume quartile stratified by functional outcomes at 90 days and discharge; the distribution of mRS scores across WMH volume quartiles at both time points; the proportion of patients in each infarct volume quartile stratified by functional outcomes at 90 days **(A)** and discharge **(B)**; and the distribution of mRS scores across infarct volume quartiles at both time points. WMH, white matter hyperintensity; mRS, modified Rankin Scale; AChA, anterior choroidal artery.

The total WMH volume was significantly higher in the 90-day poor outcome group than in the good outcome group (*p* < 0.001). Analysis of WMH volume quartiles showed that the proportion of patients in the highest quartile (Q4) was significantly higher in the poor outcome group (38.36% vs. 18.18%), while the proportion in the lowest quartile (Q1) was significantly lower (*p* < 0.001) (Table 3 and Figure 3).

**Table 3:** WMH and infarct volume characteristics in AChA territory infarction patients by 90-day mRS.

Variables	Overall *N* = 216	Discharge functional outcome		*P*-value	90-day functional outcome		*P*-value
		Poor, *N* = 93	Good, *N* = 123		Poor, *N* = 73	Good, *N* = 143	
Total WMH volume, mL	16 (6, 42)	22 (9, 52)	12 (4, 32)	<0.001	26 (12, 58)	12 (4, 30)	<0.001
WMH volume				<0.001			<0.001
Quartile 1	54 (25.00)	14 (15.05)	40 (32.52)		9 (12.33)	45 (31.47)	
Quartile 2	54 (25.00)	20 (21.51)	34 (27.64)		14 (19.18)	40 (27.97)	
Quartile 3	54 (25.00)	30 (32.26)	24 (19.51)		22 (30.14)	32 (22.38)	
Quartile 4	54 (25.00)	29 (31.18)	25 (20.33)		28 (38.36)	26 (18.18)	
PVWMH score				0.003			<0.001
Grade 0	26 (12.04)	8 (8.60)	18 (14.63)		6 (8.22)	20 (13.99)	
Grade 1	91 (42.13)	23 (24.73)	21 (17.07)		20 (27.40)	71 (49.65)	
Grade 2	55 (25.46)	29 (31.18)	62 (50.41)		25 (34.25)	30 (20.98)	
Grade 3	44 (20.37)	33 (35.48)	22 (17.89)		22 (30.14)	22 (15.38)	
DWMH score				0.027			0.003
Grade 0	76 (35.19)	23 (24.73)	53 (43.09)		16 (21.92)	60 (41.96)	
Grade 1	82 (37.96)	38 (40.86)	44 (35.77)		27 (36.99)	55 (38.46)	
Grade 2	29 (13.43)	15 (16.13)	14 (11.38)		14 (19.18)	15 (10.49)	
Grade 3	29 (13.43)	17 (18.28)	12 (9.76)		16 (21.92)	13 (9.09)	
Fazekas scale				0.046			0.006
None	25 (11.57)	9 (9.68)	16 (13.01)		7 (9.59)	18 (12.59)	
Mild	91 (42.13)	31 (33.33)	60 (48.78)		21 (28.77)	70 (48.95)	
Moderate	56 (25.93)	28 (30.11)	28 (22.76)		22 (30.14)	34 (23.78)	
Severe	44 (20.37)	25 (26.88)	19 (15.45)		23 (31.51)	21 (14.69)	
Infarct volume, mL	0.90 (0.39, 1.63)	1.44 (0.81, 2.38)	0.59 (0.30, 1.30)	<0.001	1.59 (1.17, 2.69)	0.57 (0.32, 1.18)	<0.001

Values are presented as median (IQR) or *n* (%) as appropriate. WMH, white matter hyperintensities; PVWMH, periventricular white matter hyperintensities; DWMH, deep white matter hyperintensities; mRS, modified Rankin Scale; IQR, interquartile range.

Fazekas scale assessment results indicated that, for PVWMH scores, the poor outcome group had a significantly higher proportion of grade 2–3 lesions (grade 2: 34.25% vs. 20.98%; grade 3: 30.14% vs. 15.38%) compared to the good outcome group (*p* < 0.001). DWMH scores showed a similar trend, with the poor outcome group having a significantly higher proportion of grade 2–3 lesions (grade 2: 19.18% vs. 10.49%; grade 3: 21.92% vs. 9.09%) than the good outcome group (*p* < 0.001). The total Fazekas score results were consistent, with the poor outcome group having a significantly higher proportion of severe lesions (moderate: 30.14% vs. 23.78%; severe: 31.51% vs. 14.69%) (*p* < 0.001). Infarct volume analysis showed that the lesion volume was significantly larger in the 90-day poor outcome group than in the good outcome group (*p* < 0.001).

Data analysis at discharge showed similar trends (Table 3 and Figure 3). The poor outcome group had significantly higher total WMH volume (*p* < 0.001) and a higher proportion of patients in WMH volume Q4 (*p* < 0.001). Fazekas score analysis showed that the poor outcome group had significantly higher proportions of grade 2–3 lesions in both PVWMH (*p* = 0.003) and DWMH (*p* = 0.027) compared to the good outcome group. The poor outcome group had significantly larger infarct volumes (*p* < 0.001).

Spearman correlation analysis demonstrated (Figure 2) a strong positive correlation between Fazekas visual rating assessment and quantitative WMH volume measurement (*r* = 0.84), with an even stronger correlation observed between Fazekas scores and WMH volume quartile groupings (*r* = 0.97).

### 3.4 ROC analysis of WMH and infarct volumes for outcome prediction

**Figure 4:**
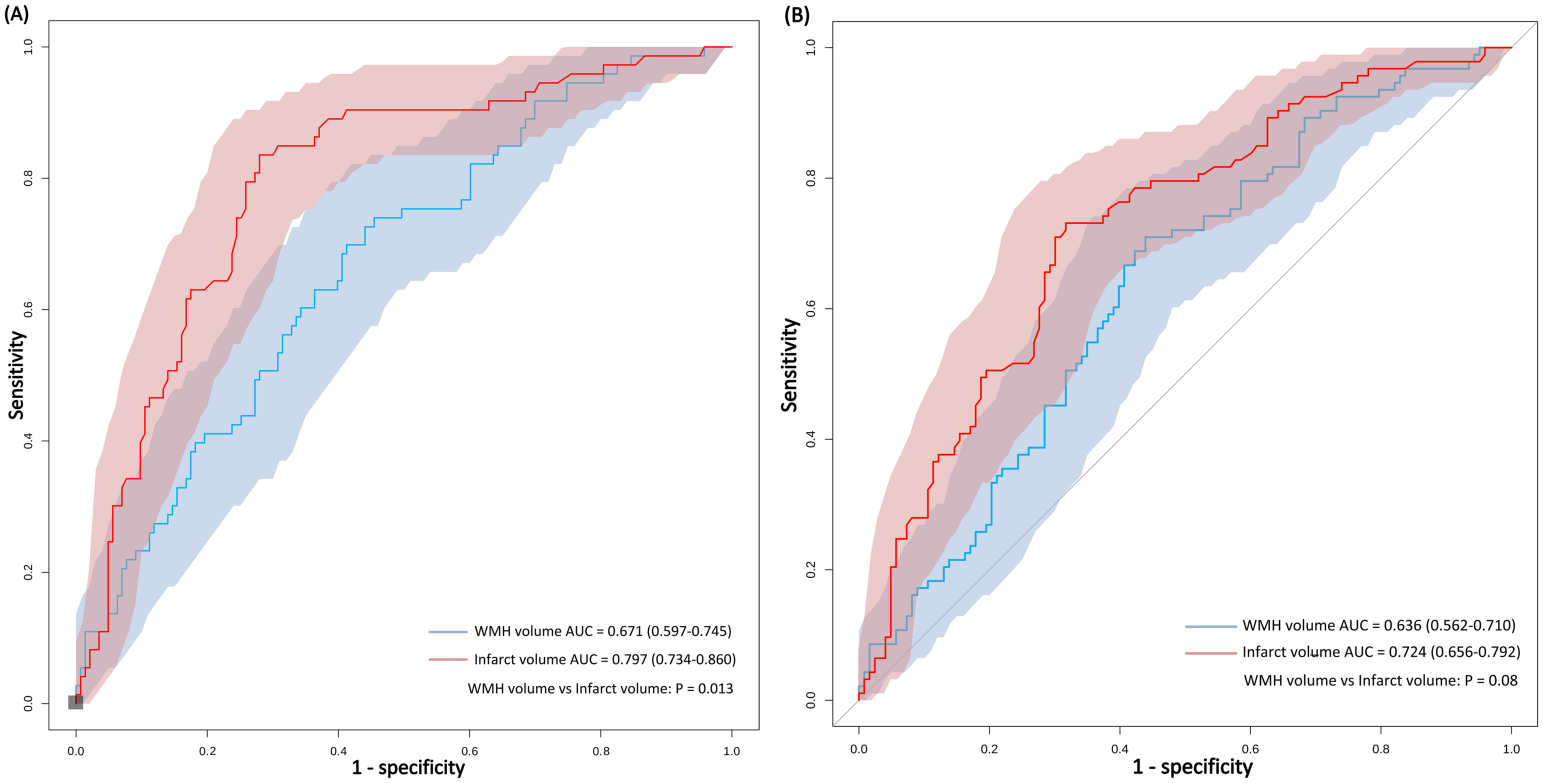
ROC curves comparing the predictive performance of WMH volume and infarct volume for functional outcomes at 90 days and discharge. Comparison of imaging predictors for stroke outcomes. ROC analysis demonstrates that infarct volume outperforms WMH volume in predicting both 90-day functional independence **(A)** and discharge functional status **(B)** in anterior choroidal artery territory stroke.

Infarct volume demonstrated a stronger predictive ability for 90-day poor outcomes compared to WMH volume (Figure 4). The AUC for infarct volume in predicting 90-day poor outcomes was 0.80, with an optimal cut-off value of 0.998 mL, yielding an accuracy of 76%, a sensitivity of 72%, and a specificity of 84%. In contrast, WMH volume showed relatively weaker predictive performance (AUC = 0.67), with an optimal cut-off value of 15.457 mL, resulting in an accuracy of 62%, a sensitivity of 59%, and a specificity of 70%. Pairwise comparison using DeLong’s test revealed statistically significant differences in discriminative performance between the two parameters (*p* = 0.013).

When predicting outcomes at discharge, the predictive ability of both parameters slightly decreased, but infarct volume (AUC = 0.72) still outperformed WMH volume (AUC = 0.64). Pairwise comparison using DeLong’s test showed no statistically significant difference in discriminative performance between the two parameters (*p* = 0.08).

### 3.5 Associated WMH burden and infarct volume with outcome

Multivariable logistic regression analysis (Table 4) showed that for each 1-mL increase in WMH volume, the risk of 90-day poor functional outcomes increased by 2% (unadjusted OR = 1.02, 95% CI: 1.00–1.03, *p* < 0.001). This association remained significant after comprehensive adjustment for confounding factors (adjusted OR = 1.02, 95% CI: 1.00–1.03, *p* = 0.032). However, no apparent dose–response relationship was observed in the WMH quartile and Fazekas score analyses. For each 1-mL increase in infarct volume, the risk of 90-day poor functional outcomes increased by 78% (adjusted OR = 1.78, 95% CI: 1.05–2.99, *p* = 0.005).

**Table 4:** Multivariable logistic regression models for 90-day outcome according to WMH burden, Fazekas Scale Grade, and infarct volume in AChA territory infarction.

Variables	Model 1		Model 2		Model 3	
	OR (95% CI)	*P*-value	OR (95% CI)	*P*-value	OR (95% CI)	*P*-value
WMH volume (continuous)	1.01 (1.00, 1.02)	<0.001	1.01 (1.00, 1.03)	0.092	1.02 (1.01, 1.03)	0.032
Infarct volume (continuous)	2.05 (1.56, 2.81)	<0.001	1.79 (1.24, 2.793)	0.004	1.78 (1.23, 2.79)	0.005
WMH volume quartiles						
Quartile 1	1.00 (Reference)		1.00 (Reference)		1.00 (Reference)	
Quartile 2	1.75 (0.68, 4.48)	0.243	1.05 (0.31, 3.59)	0.939	0.85 (0.22, 3.24)	0.810
Quartile 3	3.44 (1.40, 8.44)	0.007	1.06 (0.30, 3.75)	0.928	0.80 (0.21, 3.15)	0.754
Quartile 4	5.38 (2.20, 13.15)	<0.001	2.48 (0.62, 10.01)	0.201	3.29 (0.69, 15.71)	0.136
*P* for trend		<0.001		0.197		0.179
Fazekas scale grade						
Grade 0	1.00 (Reference)		1.00 (Reference)		1.00 (Reference)	
Grade 1	0.77 (0.29, 2.21)	0.611	0.88 (0.22, 3.70)	0.858	0.99 (0.23, 4.63)	0.991
Grade 2	1.66 (0.61, 4.88)	0.330	1.36 (0.29, 6.56)	0.697	5.85 (0.25, 6.69)	0.783
Grade 3	2.82 (1.01, 8.51)	0.054	2.91 (0.58, 15.59)	0.199	8.78 (0.63, 22.50)	0.156
*P* for trend		0.002		0.089		0.101

Values are expressed as odds ratios (ORs) with 95% confidence intervals (CIs). WMH quartiles (mL): Q1 [0–5.61], Q2 [5.61–15.9], Q3 [15.9–42], Q4 [42–181]. AChA, anterior choroidal artery; WMH, white matter hyperintensities; NIHSS, National Institutes of Health Stroke Scale; mRS, modified Rankin Scale; LDL, low-density lipoprotein.

In contrast, the analysis of functional outcomes at discharge showed weaker associations (Table 5). WMH volume was associated with poor outcomes in the unadjusted model (OR = 1.01, 95%CI: 1.01–1.02, *p* = 0.011) but lost statistical significance after adjusting for confounding factors. Similarly, the risk of poor outcomes in the highest WMH burden group lost statistical significance after adjustment. The association between infarct volume and outcomes at discharge also lacked statistical significance after adjustment.

**Table 5:** Multivariable logistic regression analysis of WMH burden and infarct volume associated with discharge functional outcome in patients with anterior choroidal artery territory infarction.

Variables	Model 1		Model 2		Model 3	
	OR (95% CI)	*P*-value	OR (95% CI)	*P*-value	OR (95% CI)	*P*-value
WMH volume (continuous)	1.01 (1.01, 1.02)	0.011	1.00 (0.99, 1.02)	0.579	1.01 (0.99, 1.02)	0.487
Infarct volume (continuous)	1.67 (1.31, 2.22)	<0.001	1.36 (0.94, 1.99)	0.094	1.31 (0.89, 1.92)	0.155
WMH volume quartiles						
Quartile 1	1.00 (Reference)		1.00 (Reference)		1.00 (Reference)	
Quartile 2	1.68 (0.74, 3.82)	0.216	0.93 (0.28, 3.01)	0.897	0.76 (0.21, 2.78)	0.681
Quartile 3	3.57 (1.59, 8.04)	0.002	1.17 (0.33, 4.17)	0.814	0.89 (0.23, 3.48)	0.867
Quartile 4	3.31 (1.47, 7.45)	0.004	1.27 (0.30, 5.39)	0.747	1.24 (0.26, 6.03)	0.787
*P* for trend		<0.001		0.449		0.593
Fazekas scale grade						
Grade 0	1.00 (Reference)		1.00 (Reference)		1.00 (Reference)	
Grade 1	0.92 (0.37, 2.39)	0.857	1.07 (0.26, 4.61)	0.923	1.22 (0.27, 5.74)	0.796
Grade 2	1.78 (0.68, 4.84)	0.245	1.53 (0.32, 7.73)	0.598	1.49 (0.27, 8.33)	0.642
Grade 3	2.34 (0.86, 6.63)	0.100	2.80 (0.52, 16.07)	0.236	3.13 (0.50, 20.90)	0.226
*P* for trend		0.010		0.156		0.191

Values are expressed as odds ratios (ORs) with 95% confidence intervals (CIs). WMH quartiles (mL): Q1 [0–5.61], Q2 [5.61–15.9], Q3 [15.9–42], Q4 [42–181]. AChA, anterior choroidal artery; WMH, white matter hyperintensities; NIHSS, National Institutes of Health Stroke Scale; mRS, modified Rankin Scale; LDL, low-density lipoprotein.

### 3.6 Non-linear association analysis and threshold effect

**Figure 5:**
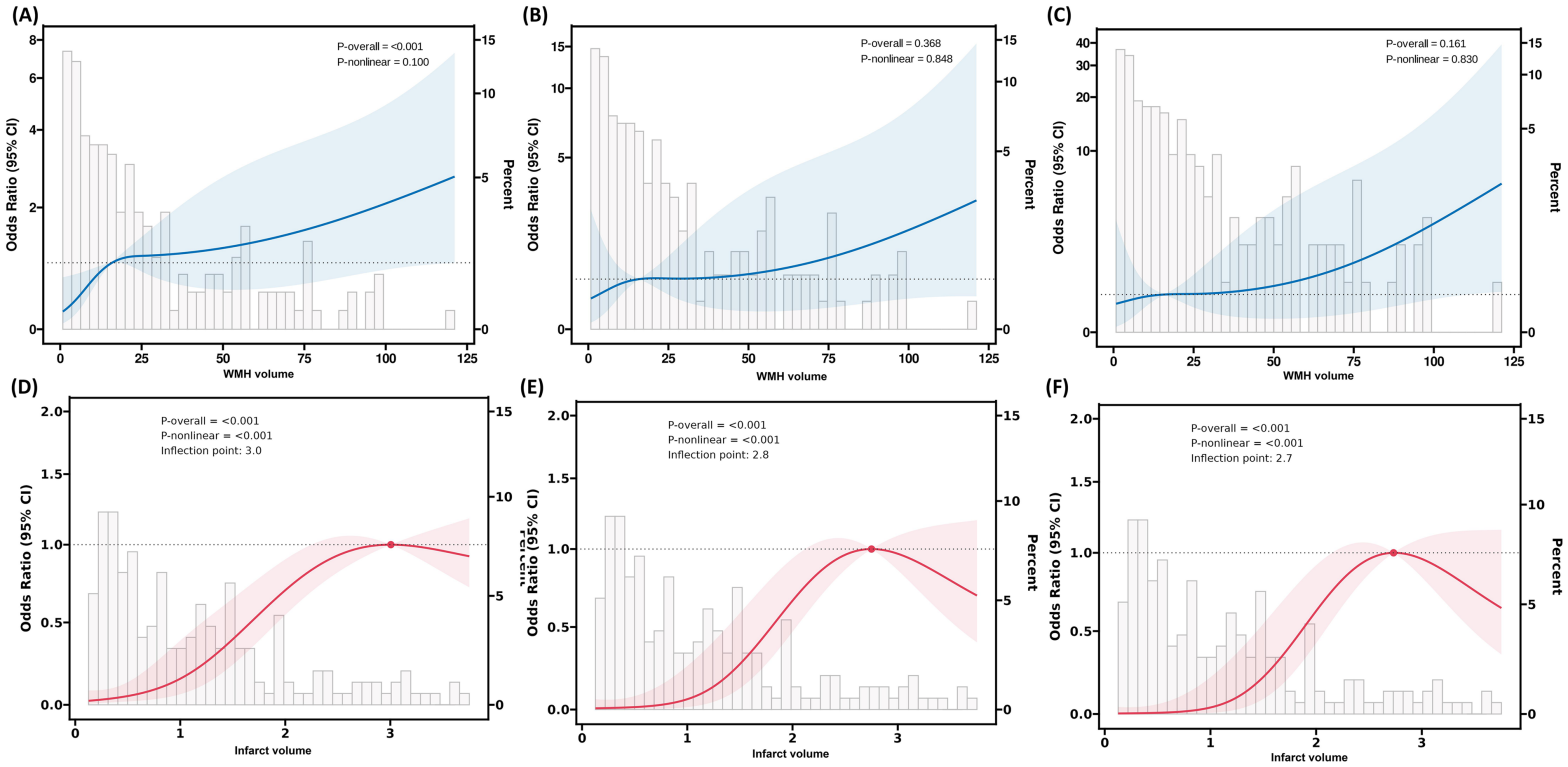
RCS analysis of the association between WMH volume, infarct volume, and 90-day functional outcomes in patients with AChA territory infarction. **(A–C)** RCS plots illustrating the relationship between WMH volume and the odds of poor functional outcomes at 90 days in three stepwise-adjusted models. No significant non-linear association was observed between WMH volume and 90-day functional outcomes in any of the models (*P*-non-linear > 0.05). **(D–F)** RCS plots demonstrating the association between infarct volume and the odds of poor functional outcomes at 90 days in the same three stepwise-adjusted models. The inflection point (knot) was located at an infarct volume of 2.7 mL, indicating a threshold effect. RCS, restricted cubic spline; WMH, white matter hyperintensity; AChA, anterior choroidal artery; mRS, modified Rankin Scale.

After fully adjusting for confounding factors, RCS analysis revealed a significant non-linear association between infarct volume and 90-day functional outcomes (Figure 5). The RCS curve showed a clear inflection point at 2.7 mL, exhibiting an inverted U-shaped distribution (*P*-non-linear<0.001). Piecewise regression analysis (Table 6) indicated that when infarct volume was <2.7 mL, each 1-mL increase was significantly associated with 90-day functional outcomes (adjusted OR = 5.31, 95% CI: 3.07–9.73; standardized OR = 3.03, 95% CI: 2.11–4.53; both *p* < 0.001). However, after full adjustment, no non-linear relationship was observed between WMH volume and 90-day functional outcomes.

**Table 6:** Segmented regression analysis of infarct volume for functional outcomes based on restricted cubic spline-derived thresholds in anterior choroidal artery territory infarction.

Variables	OR (95% CI)	*P*-value	OR per SD (95% CI)	*P*-value
Infarct volume				
<2.7 ml	5.31 (3.07, 9.73)	<0.001	3.03 (2.11, 4.53)	<0.001
≥2.7 ml	0.88 (0.56, 1.42)	0.58	0.79 (0.34, 1.90)	0.580

Values are presented as odds ratios (ORs) with 95% confidence intervals (CIs). OR per SD represents the odds ratio per 1-standard deviation increase. Adjusted for age, Hypertension, baseline NIHSS score, baseline mRS score, anatomical involvement (corona radiata, lateral thalamus), lesion size and antiplatelet therapy, laboratory parameters (neutrophil, lymphocyte, total protein, albumin, LDL cholesterol, fasting blood glucose, and fibrinogen).

## 4 Discussion

This study is the first to systematically evaluate the value of WMH burden and infarct volume in predicting functional outcomes in patients with AChA territory infarction, revealing several key findings. First, after comprehensively adjusting for confounding factors, both WMH burden and infarct volume were independently associated with 90-day poor functional outcomes, with infarct volume demonstrating superior predictive performance (AUC: 0.80 vs. 0.67). Second, we identified through RCS analysis an exploratory potential clinical threshold (2.7 mL). When infarct volume was below this threshold, each 1-mL increase was associated with a 5.31-fold increased risk of poor outcomes, while this association plateaued beyond the threshold.

These findings are consistent with previous research. Multiple large-scale studies have confirmed that WMH burden is an independent predictor of 3-month functional outcomes, 1-year mortality, and long-term stroke recurrence in ischemic stroke patients (Bonkhoff et al., 2022; Dimaras et al., 2023; Gwak et al., 2024; Hong et al., 2021; Ryu et al., 2017; Ryu et al., 2019; Wu et al., 2021). In a study involving 890 patients with acute ischemic stroke (AIS), Hong et al. found that WMH burden was closely related to long-term functional outcomes (*β* = 0.104, *p* < 0.01) (Hong et al., 2021). Helenius et al. (2017), using the Fazekas scale, demonstrated that moderate-to-severe WMH burden (OR = 2.28) and larger infarct volume (OR = 1.98) were independently associated with 90-day poor outcomes in patients with subcortical infarction.

The WMH may affect neurological function in AIS patients during both the acute phase and recovery period through multiple pathophysiological mechanisms. In the acute phase, WMH primarily exacerbates ischemic injury through vascular dysfunction and hemodynamic alterations. On one hand, WMH impairs collateral circulation function, which manifests in patients with large vessel occlusion as larger infarct core volumes on CT perfusion imaging, lower collateral circulation grades, and faster infarct growth rates, significantly shortening the survival time of the ischemic penumbra (He et al., 2024). On the other hand, structural abnormalities of perforating arteries in WMH regions, including vessel wall thickening, hyaline degeneration, and vascular narrowing, elongation, and tortuosity, result in reduced regional cerebral blood flow and decreased cerebral oxygen utilization, thereby compromising the brain’s tolerance to new ischemic insults (O'Sullivan et al., 2002; Juttukonda et al., 2022). Furthermore, the hypercoagulable state associated with WMH not only affects the efficacy of reperfusion therapy but may also induce the “no-reflow phenomenon,” characterized by incomplete restoration of microcirculatory blood flow following vessel recanalization (Tomimoto et al., 2001).

During the recovery period, WMH primarily affects neurological recovery by disrupting white matter fiber network integrity and causing cerebral metabolic dysfunction. Damage to white matter fiber connectivity networks in patients with high WMH burden can lead to interrupted information transmission pathways, impairing neuroplasticity and brain reserve capacity, thereby hindering neurological recovery (Etherton et al., 2019; Lawrence et al., 2014). Concurrently, cerebral metabolic abnormalities represent another important mechanism. 18F-fluorodeoxyglucose positron emission tomography studies have confirmed significantly reduced glucose metabolism in white matter regions of WMH patients (Haight et al., 2013; Pascual et al., 2010). Longitudinal studies further reveal that WMH patients exhibit reduced metabolic levels in lesioned regions at baseline, with follow-up demonstrating progressive metabolic decline in normal-appearing white matter regions, while advanced WMH areas show evident structural destruction, suggesting that metabolic dysfunction may serve as an important mediator of WMH progression and limited functional recovery (Jiaerken et al., 2019).

Interestingly, although both WMH burden robustly predicted 90-day functional outcomes, their associations with discharge outcomes were markedly weaker. Further analysis revealed (Table 3) that patients with lower WMH burden (first and second quartiles) had significantly higher rates of recovery from functional dependence at discharge to functional independence at 90 days compared with those with higher burden (third and fourth quartiles). This may suggest that lower imaging burden could indicate greater early rehabilitation potential, and this differential recovery pattern reinforces the importance of WMH burden and infarct volume as prognostic predictors. This hypothesis is supported by previous large-scale studies. Ryu et al. (2017) found in a study involving 5,035 patients with ischemic stroke that, as WMH burden increased, the likelihood of functional improvement gradually decreased (adjusted ORs for the 3rd, 4th, and 5th quintiles were 0.81, 0.81, and 0.67, respectively), showing a significant dose–response relationship. Hong et al. discovered that high WMH burden significantly reduced patients’ chances of achieving complete functional independence (compared to the low burden group: OR = 0.40, 95% CI: 0.25–0.63, *p* < 0.01; compared to the moderate burden group: OR = 0.61, 95% CI: 0.42–0.87, *p* < 0.01) (Hong et al., 2021). Based on this observation, clinicians may consider implementing more intensive early rehabilitation interventions for patients who demonstrate poor functional status at discharge but harbor relatively low WMH burden, with the aim of maximizing their neurological recovery potential. Nevertheless, the efficacy of such rehabilitation strategies warrants validation through well-designed prospective randomized controlled trials.

The findings of this study provide valuable imaging-based insights for the clinical management of patients with AChA territory infarction. From a pathophysiological perspective, we have systematically elucidated, for the first time, the independent predictive roles of WMH burden and infarct volume on functional outcomes in AChA infarction patients. Through RCS analysis, we have exploratorily identified a potential clinical threshold for infarct volume, providing quantitative imaging evidence for a deeper understanding of prognostic patterns in AChA infarction. It is important to emphasize that clinical management recommendations based on this exploratory threshold should be approached with caution. For patients with infarct volumes approaching or exceeding 2.7 mL, clinicians may consider intensifying early monitoring and implementing more aggressive rehabilitation strategies; however, specific management approaches should be individualized based on the patient’s overall clinical status. Regarding predictive model optimization, integrating WMH burden and acute infarct volume into existing stroke prognostic assessment systems holds promise for enhancing overall model performance, though this hypothesis requires validation in independent cohorts.

This study has several important limitations that must be fully considered when interpreting the results. First, the inherent limitations of retrospective cohort study design include potential selection bias and information bias. Although strict inclusion and exclusion criteria were applied and major confounding factors were adjusted for in multivariable models, the strategy of selecting adjustment variables based on statistical significance may have overlooked important confounders (including key vascular risk factors and other acute treatment variables), thereby affecting the accurate estimation of association strength. Second, the relatively limited follow-up period represents another significant limitation. This study evaluated only 90-day functional outcomes, whereas neurological recovery is a continuous, dynamic process. Longer-term follow-up (such as 6 months or 1 year) might reveal different prognostic patterns and changes in the role of risk factors. Third, external validity and threshold robustness constitute core limitations of this study. The study population was primarily derived from two stroke centers in China, with relatively homogeneous geographic and ethnic composition. More importantly, the 2.7-mL threshold identified in this study has not been validated for statistical robustness through methods such as bootstrap resampling or cross-validation, posing a risk of overfitting to the current dataset. Therefore, this threshold should be strictly regarded as an exploratory finding, and its reproducibility and generalizability across different populations, healthcare systems, and imaging technology conditions remain to be verified. Nevertheless, our study provides a potential direction for establishing universal prognostic prediction thresholds in the future. Future research will require large-sample, multicenter studies employing standardized imaging protocols and rigorous statistical validation methods to establish prognostic prediction thresholds with universal applicability and clinical utility, ultimately achieving effective translation from scientific discovery to clinical application.

## 5 Conclusion

In conclusion, this study found that both WMH burden and infarct volume are independent predictors of 90-day functional outcomes in patients with AChA territory infarction, with infarct volume demonstrating superior predictive value. We also revealed a non-linear association between infarct volume and prognosis, identifying an exploratory potential clinical threshold. When infarct volume is below this threshold, the risk of poor outcomes increases substantially with each 1-mL increase in volume. These findings provide new imaging-based evidence for early risk stratification and individualized treatment decision-making in patients with AChA infarction.

## Data Availability

The datasets used and/or analyzed during the current study are available from the corresponding author upon reasonable request. Requests to access the datasets should be directed to Renjing Zhu, zhurenjing@163.com.

## Footnotes

^1^ https://www.statistical-modelling.de/lst.html

^2^ https://stroke-maps.github.io/anterior-choroidal
