# Supplementary material for: White matter hyperintensity burden and infarct volume predict functional outcomes in anterior choroidal artery stroke: a multimodal MRI study
Source: Front Neurosci. 2025 Aug 6;19:1625882. doi: 10.3389/fnins.2025.1625882 (PMC12365522; doi:10.3389/fnins.2025.1625882)
Supplement: Supplementary file 1 [file Table_1.docx]

| **Supplementary Table S1.** Multicollinearity Diagnostics for Multivariable Models | | | | | | | | | | |
| --- | --- | --- | --- | --- | --- | --- | --- | --- | --- | --- |
| **Term** | **VIF^a^** | **Tolerance^a^** | **VIF^b^** | **Tolerance^b^** | **VIF^c^** | **Tolerance^c^** | **VIF^d^** | **Tolerance^d^** | **VIF^e^** | **Tolerance^e^** |
| WMH volume | 1.30176 | 0.7681906 | - | - | - | - | - | - | - | - |
| Infarct volume | - | - | 1.480735 | 0.6753404 | - | - | - | - | - | - |
| WMH volume quartiles | - | - | - | - | 2.177767 | 0.4591859 | - | - | - | - |
| Fazekas scale grade | - | - | - | - | - | - | 1.840488 | 0.5433342 | - | - |
| Age, years | 1.509671 | 0.662396 | 1.438131 | 0.6953469 | 1.817661 | 0.5501575 | 1.681161 | 0.5948269 | 1.522452 | 0.6568352 |
| Hypertension | 1.156085 | 0.864988 | 1.158869 | 0.8629104 | 1.184081 | 0.8445366 | 1.227723 | 0.8145162 | 1.241627 | 0.805395 |
| Baseline NIHSS score | 2.418064 | 0.413554 | 2.436611 | 0.4104062 | 2.441342 | 0.4096107 | 2.453919 | 0.4075115 | 2.636868 | 0.3792378 |
| Baseline mRS score | 2.43774 | 0.4102159 | 2.509489 | 0.3984874 | 2.415434 | 0.4140043 | 2.444903 | 0.4090141 | 3.421715 | 0.2922511 |
| Lesion size | 1.301859 | 0.7681322 | 1.448968 | 0.6901461 | 1.265679 | 0.7900897 | 1.300795 | 0.7687605 | 1.588183 | 0.6296502 |
| **Involved anatomical regions** |  |  |  |  |  |  |  |  |  |  |
| Corona radiata | 1.185828 | 0.8432929 | 1.253922 | 0.797498 | 1.215101 | 0.8229767 | 1.187883 | 0.8418339 | 1.520964 | 0.6574777 |
| Lateral thalamus | 1.145126 | 0.8732666 | 1.231785 | 0.8118299 | 1.192902 | 0.8382921 | 1.166165 | 0.8575114 | 1.350206 | 0.7406275 |
| Antiplatelet therapy | 1.240149 | 0.8063544 | 1.251521 | 0.7990275 | 1.282064 | 0.7799924 | 1.230625 | 0.8125951 | 1.5598 | 0.6411078 |
| Neutrophils | 1.236288 | 0.808873 | 1.236694 | 0.8086074 | 1.252662 | 0.7983 | 1.238631 | 0.8073431 | 1.306841 | 0.7652043 |
| Lymphocytes | 1.162558 | 0.8601718 | 1.144346 | 0.8738617 | 1.242512 | 0.8048213 | 1.23402 | 0.8103595 | 1.334567 | 0.7493069 |
| Total protein | 1.981354 | 0.5047054 | 1.767712 | 0.5657029 | 1.94926 | 0.5130152 | 1.967103 | 0.5083619 | 1.735903 | 0.5760691 |
| Albumin | 2.415237 | 0.4140381 | 2.152856 | 0.4644992 | 2.3289 | 0.4293872 | 2.339081 | 0.4275183 | 2.073608 | 0.4822513 |
| LDL cholesterol | 1.354006 | 0.7385493 | 1.406063 | 0.7112055 | 1.373945 | 0.7278314 | 1.394684 | 0.7170085 | 1.428147 | 0.7002083 |
| FBG | 1.117882 | 0.8945492 | 1.141031 | 0.8764003 | 1.167032 | 0.8568742 | 1.151952 | 0.8680919 | 1.155297 | 0.8655782 |
| Fibrinogen | 1.158566 | 0.863136 | 1.188336 | 0.8415129 | 1.168883 | 0.8555173 | 1.169553 | 0.8550276 | 1.357807 | 0.7364816 |
| Footnotes: ^a^ Model including WMH volume as continuous variable. ^b^ Model including infarct volume as continuous variable. ^c^ Model including WMH volume as quartiles. ^D^ Model including Fazekas scale grade. ^e^ Model including infarct volume as quartiles. VIF >10 or tolerance <0.1 indicates potential multicollinearity concerns. All models showed acceptable multicollinearity levels (VIF <10, tolerance >0.1). WMH, white matter hyperintensity; VIF, variance inflation factor; NIHSS, National Institutes of Health Stroke Scale; mRS, modified Rankin Scale; LDL, low-density lipoprotein; FBG, fasting blood glucose. | | | | | | | | | | |
